# Teratocarcinomas Arising from Allogeneic Induced Pluripotent Stem Cell-Derived Cardiac Tissue Constructs Provoked Host Immune Rejection in Mice

**DOI:** 10.1038/srep19464

**Published:** 2016-01-14

**Authors:** Ai Kawamura, Shigeru Miyagawa, Satsuki Fukushima, Takuji Kawamura, Noriyuki Kashiyama, Emiko Ito, Tadashi Watabe, Shigeo Masuda, Koichi Toda, Jun Hatazawa, Eiichi Morii, Yoshiki Sawa

**Affiliations:** 1 Department of Cardiovascular Surgery, Osaka University Graduate School of Medicine, Suita, Osaka, Japan; 2 Department of Nuclear Medicine and Tracer Kinetics, Osaka University Graduate School of Medicine, Suita, Osaka, Japan; 3 Immunology Frontier Research Center, Osaka University, Suita, Osaka, Japan; 4 Department of Diagnostic Pathology, Osaka University Graduate School of Medicine, Suita, Osaka, Japan

## Abstract

Transplantation of induced pluripotent stem cell-derived cardiac tissue constructs is a promising regenerative treatment for cardiac failure: however, its tumourigenic potential is concerning. We hypothesised that the tumourigenic potential may be eliminated by the host immune response after allogeneic cell transplantation. Scaffold-free iPSC-derived cardaic tissue sheets of C57BL/6 mouse origin were transplanted into the cardiac surface of syngeneic C57BL/6 mice and allogeneic BALB/c mice with or without tacrolimus injection. Syngeneic mice and tacrolimus-injected immunosuppressed allogeneic mice formed teratocarcinomas with identical phenotypes, characteristic, and time courses, as assessed by imaging tools including ^18^F-fluorodeoxyglucose-positron emission tomography. In contrast, temporarily immunosuppressed allogeneic mice, following cessation of tacrolimus injection displayed diminished progression of the teratocarcinoma, accompanied by an accumulation of CD4/CD8-positive T cells, and finally achieved complete elimination of the teratocarcinoma. Our results indicated that malignant teratocarcinomas arising from induced pluripotent stem cell-derived cardiac tissue constructs provoked T cell-related host immune rejection to arrest tumour growth in murine allogeneic transplantation models.

Cardiac failure is a leading cause of mortality worldwide. Although heart transplantation and ventricular assist device implantation can improve the survival of patients with end-stage cardiac failure, the clinical indication of these therapies is substantially limited^1^. Regenerative therapy using derivatives of induced pluripotent stem cells (iPSCs) may be an alternative method to treat end-stage cardiac failure^2^, because transplanted iPSC-derived cardiomyocytes (iPSC-CMs) on the heart can synergistically contract with native cardiomyocytes to generate mechanical force in animal models of ischemic cardiac failure^3^. However, the tumourigenic potential of transplanted iPSC-derivatives is concerning^4^.

Transplantation of iPSC-derivatives, regardless of the target phenotype or site of transplantation, may cause teratoma/teratocarcinoma formation, which theoretically originates from either (1) residual undifferentiated iPSCs in the derivatives and/or (2) tumourigenic mutation of the genome/epigenome upon reprogramming or during the differentiation process^5^. As the use of “banked” iPSCs, which were screened for tumourigenicity in advance, would prevent genome/epigenome mutation-related tumour formation^6^, transplantation of allogeneic iPSCs from the “bank” is clinically warranted despite the need for immunosuppressive therapy targeting allograft antigens^7^,^8^. In addition, regulation of the host-immune response against the allograft could treat tumours arising from iPSC-derivatives. Importantly, Itakura *et al*. reported that glioma formation from xenogeneic iPSC-derivatives in the spinal cord was inhibited by activation of the xenotransplantation-related immune response^9^. We, therefore, hypothesised that tumourigenic potential of iPSC-derived cardiac tissue constructs may be affected by the host immune response following allogeneic cell transplantation in the heart.

To test this hypothesis, we generated an immunosuppressed allogeneic transplantation model, which is immunologically equivalent to the syngeneic model. In addition, we examined the time course of tumour formation following iPSC-CM transplantation in the heart using the latest clinical imaging tools for tumour diagnosis, including computed tomography (CT), enhanced magnetic resonance imaging (MRI), and ^18^F-fluorodeoxyglucose (FDG)-positron emission tomography (PET) in the murine syngeneic and allogeneic transplantation models. Moreover, the immunological reaction associated with tumour formation was explored by modulating immunosuppressant therapy during the early phase using diagnostic imaging in the allogeneic transplantation model.

## Results

### Luminescence active tumuor formation from miPSC-CMs in a syngeneic model

**Figure 1:**
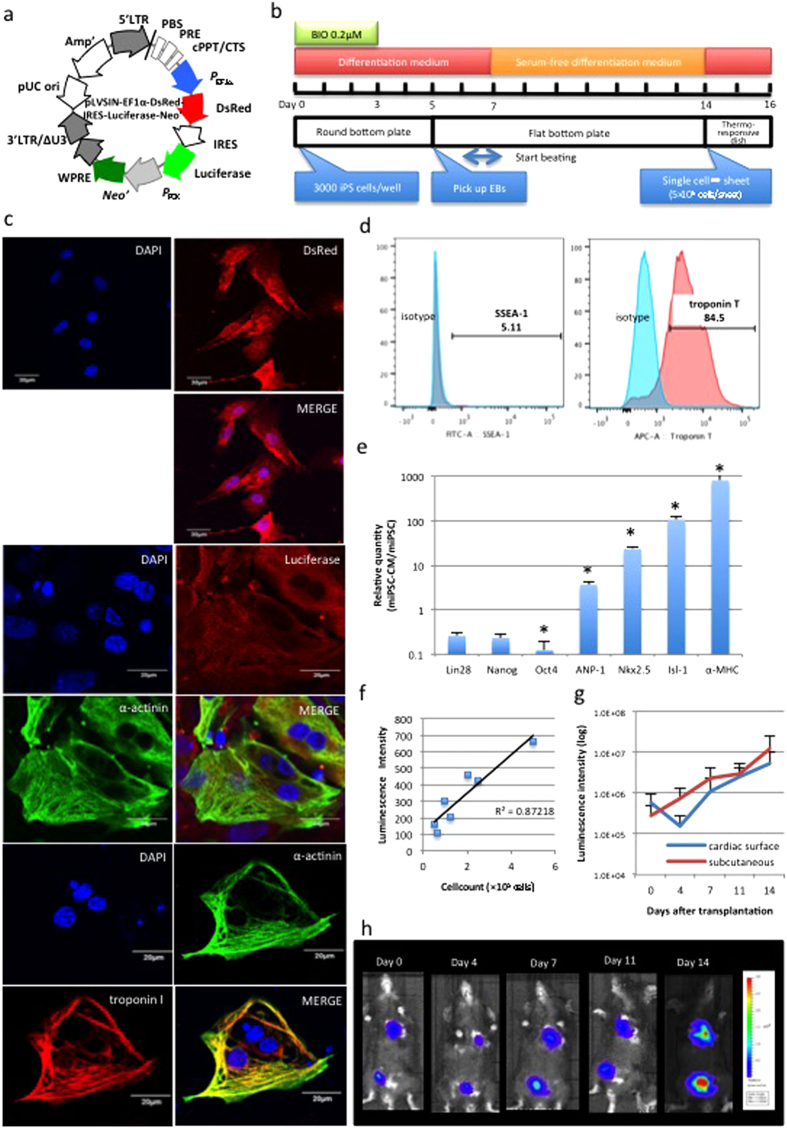
Generation of DsRed-Luciferase-miPSC-derived cardiac tissue constructs for creating bioluminescent tumours in a syngeneic transplantation model. (**A**) DsRed-Luciferase-miPSCs were generated using the miPSC 959A2-1 line transfected with a recombinant lentiviral vector containing the *DsRed, Luciferase*, and *neomycin resistance* genes. (**B**) The cardiomyogenic differentiation process. (**C**) Representative confocal images with immunohistolabelling revealed expression of DsRed, Luciferase, troponin I and α-actinin in the cytoplasm of each beating cell in the DsRed-Luciferase-derived cardiac tissue constructs. (**D**) A representative flow cytometry histogram demonstrated that 5.11% and 84.5% of the DsRed-Luciferase-miPSC-derived cardiac tissue constructs were positive for SSEA-1 and troponin T, respectively. (**E**) DsRed-Luciferase-miPSC-derived cardiac tissue constructs displayed reduced Lin28, Oct4, and Nanog and increased ANP-1, Nkx2.5, Isl-1, and *α*-MHC expression compared with that of undifferentiated DsRed-Luciferase-miPSCs (* p < 0.05) by real-time PCR. (**F**) Luminescence intensity and total cell number were positively correlated *in vitro*. (**G**) The DsRed-Luciferase-miPSC-derived cardiac tissue construct-sheets were transplanted into the cardiac surface and the abdominal subcutaneous tissue of syngeneic C57BL/6 mice (n = 4). The quantitative BLI study indicated that luminescence intensity was similar regardless of whether cell-sheets were transplanted into the heart or into the subcutaneous tissue. (**H**) Representative serial images of the BLI study in one mouse. Abbreviations: EB; embryonic body, BIO; 6-bromoindirubin-3′-oxime, ANP; atrial natriuretic peptide, MHC; myosin heavy chain, BLI; bioluminescence imaging.

The murine iPSC (miPSC) line, 959A2-1, generated from C57BL/6 mouse embryonic fibroblasts (gift from Dr. Okita and Professor Yamanaka, Kyoto University, Japan) was used in this study^10^. A pLVSIN-EF1*α*-DsRed-IRES-Luciferase-Neo lentiviral vector was transfected into 959A2-1 cells, which were then treated with neomycin to establish a miPSC line that stably expressed DsRed and Luciferase (DsRed-Luciferase-miPSC; Fig. 1a).

Cardiomyogenic differentiation of DsRed-Luciferase-miPSCs was induced according to the schema in Fig. 1b. On day 6 or 7, cells spontaneously started regular beating. After 14 days of differentiation process, immunohistolabelling of the beating cell clusters demonstrated that all cells expressed DsRed and Luciferase in the cytoplasm, and that troponin I and *α*-actinin were labelled in the cytoplasm, indicating well-aligned sarcomere structure (Fig. 1c). On the other hand, 67–86% of the cells were positive for troponin T, while 3–9% of the cells were positive for stage-specific embryonic antigen (SSEA)-1, one of the major undifferentiated cell surface markers, as assessed by flow cytometry (Fig. 1d). The cell populations, which was called “DsRed-Luciferase-miPSC-derived cardiac tissue constructs” in this manuscript, displayed significantly higher *atrial natriuretic peptide (ANP)-1, Nkx2.5*, *Isl-1*, and *α-myosin heavy chain (MHC)* expression and significantly lower *Oct4* expression than those observed in undifferentiated DsRed-Luciferase-miPSCs, whereas *Lin28* and *Nanog* were still detected in DsRed-Luciferase-miPSC-derived cardiac tissue constructs with lower levels without statistically significant difference, reflecting the presence of remaining undifferentiated iPSCs after the cardiac differentiation process.

The luminescence intensity of the DsRed-Luciferase-miPSC-derived cardiac tissue constructs was positively correlated with the cell count *in vitro* (Fig. 1f). Additionally, the cell-sheets were transplanted into the cardiac surface and the abdominal subcutaneous tissue of syngeneic C57BL/6 mice (n = 4) to examine the feasibility of bioluminescence imaging (BLI) of the heart. Photons were clearly detected in both locations without significant differences in the time course (Fig. 1g,h). By day 14, all mice developed large tumours in the chest cavity and the subcutaneous tissue. Thus, the use of the DsRed-Luciferase-miPSC cell-line was warranted.

### Teratocarcinoma formation in the immunosuppressed, but not in the immunocompetent allogeneic transplantation models

**Figure 2:**
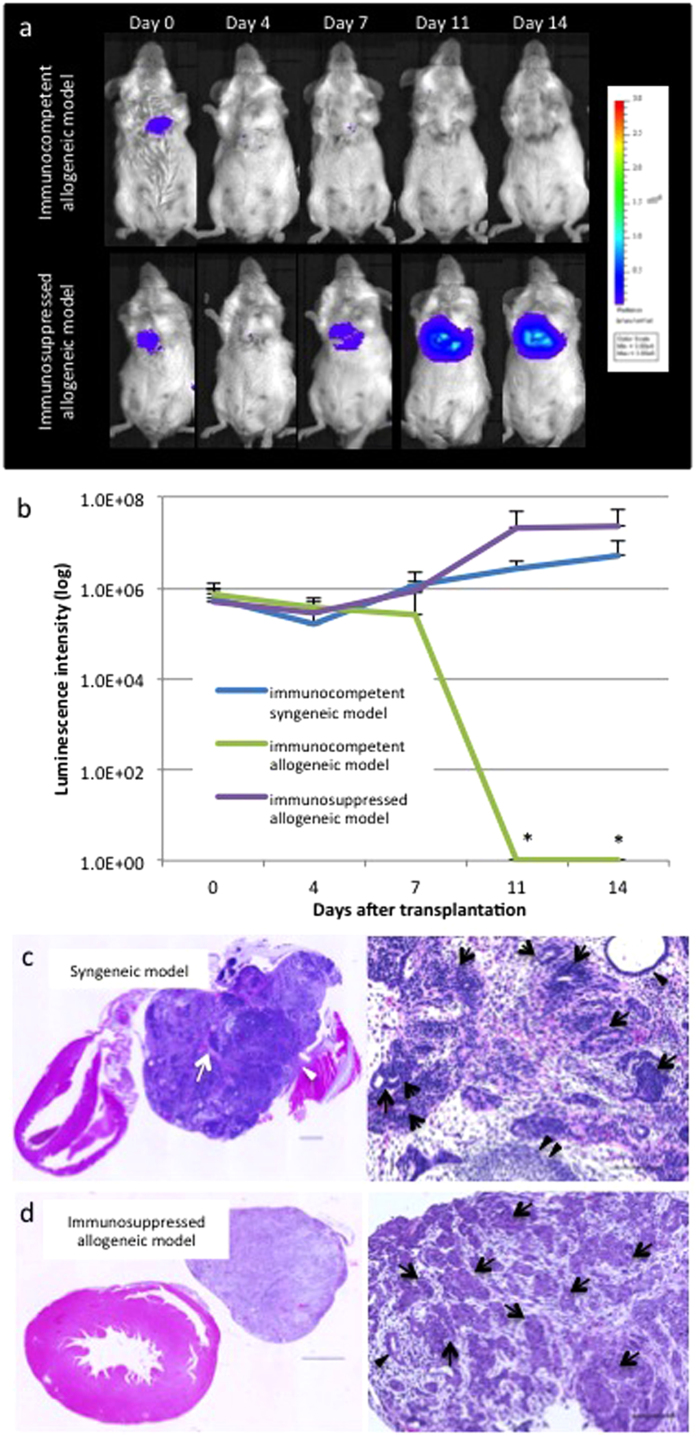
Teratocarcinoma formation in an immunosuppressed allogeneic transplantation model. (**A**) Representative serial images of the BLI study in one immunocompetent and one immunosuppressed allogeneic mouse. (**B**) Quantitative analysis of BLI displayed a gradual increase of photons in the immunosuppressed allogeneic mice (n = 3) identically to those of syngeneic mice, whereas there was a gradual decrease and ultimate elimination (*) in the immunocompetent allogeneic mice (n = 3). (**C**) Representative images of a teratocarcinoma that contains three germ-line structures (cartilage [black double arrowhead], gland tissue [black arrowhead] and immature neural tube [black arrow]), central necrosis (white arrow) and invasion into adjacent intercostal muscle (white arrowhead) in the immunocompetent syngeneic model. (**D**) Representative images of a teratocarcinoma that contains two germ-line structures (gland tissue [black arrowhead] and immature neural tube [black arrow]) in the immunosuppressed allogeneic model. Scare bars = 1000 μm (left) and 100 μm (right). Abbreviation: BLI; bioluminescence imaging.

DsRed-Luciferase-miPSC-derived cardiac tissue-sheets were transplanted into the cardiac surface of control (immunocompetent allogeneic model; n = 3) and tacrolimus-treated BALB/c mice (immunosuppressed allogeneic model; n = 3). The immunosuppressed allogeneic mice, whose blood concentration of tacrolimus was 134.0 ± 24.5 ng/ml on the third day following transplantation of tacrolimus infusion pumps and subsequently remained stable, displayed an identical increase of photons to that of the immunocompetent syngeneic mice by day 14, as assessed by BLI. In contrast, immunocompetent allogeneic mice displayed a decrease of photons by day 14 (Fig. 2a,b). All immunosuppressed allogeneic mice developed huge tumours in the chest cavity by day 14, which were histologically diagnosed as teratocarcinomas and identical to those of immunocompetent syngeneic mice (Fig. 2c,d).

### ^18^F-FDG PET, as a sharp diagnostic imaging tool of teratocarcinoma formation

**Figure 3:**
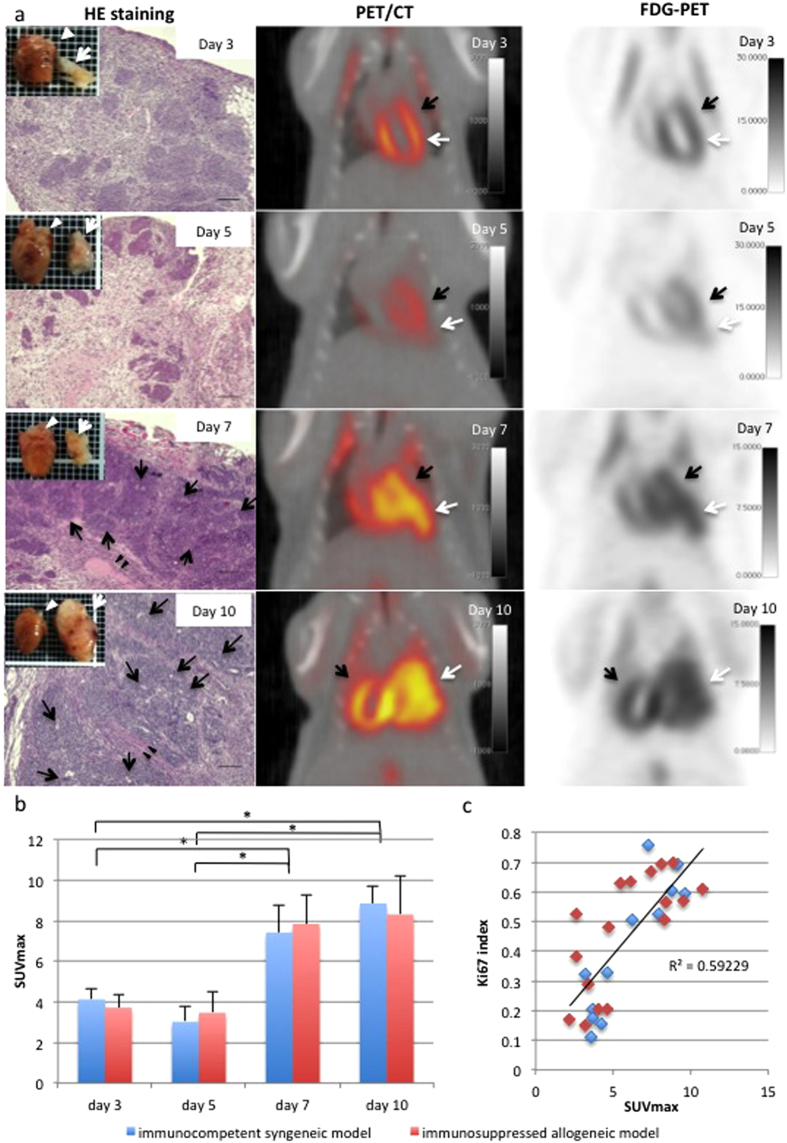
A time course of teratocarcinoma formation detected by ^18^F-FDG PET studies. (**A**) Representative images of histological analysis using HE staining (left) and ^18^F-FDG PET/CT imaging (middle and right) in syngeneic models. Histologically, immature neural tube formation (black arrow) or central necrosis (black arrow head) was detected from day 7 onwards, and the tumour was diagnosed as a teratocarcinoma (scare bars = 100 μm). The teratocarcinoma (white arrow) exceeded the host’s heart (white arrowhead) in size by day 10. ^18^F-FDG PET images (right) and fusion images of PET/CT study (middle) revealed high ^18^F-FDG uptake by the teratocarcinoma (white arrow) adjacent to the physiological accumulation of ^18^F-FDG in the host’s heart (black arrow). (**B**) The SUVmax displayed a similar increase from day 7 onwards (*p < 0.05) in both the syngeneic (n = 3; each day) and immunosuppressed allogeneic model (n = 4; day 3, 5 and 7, n = 5; day 10). (**C**) The SUVmax and the Ki67 index were positively correlated. Abbreviations: SUV; standardised uptake value, HE; hematoxylin-eosin.

Immunocompetent syngeneic (n = 12) and immunosuppressed allogeneic mice (n = 17) that received the DsRed-Luciferase-miPSC-derived cardiac tissue constructs transplant were serially examined using ^18^F-FDG-PET, CT, and enhanced MRI, to compare the characteristics and time courses of teratocarcinoma formation. Increased uptake of ^18^F-FDG at the cardiac surface was detected from day 7 onwards, compared with that on days 3 and 5, in the immunocompetent syngeneic model (Fig. 3a). The maximum standardised uptake values (SUVmax) were significantly higher on days 7 and 10, compared with those on days 3 and 5 (p < 0.05). The ^18^F-FDG-PET study followed by the SUVmax analysis failed to reveal differences between the immunosuppressive allogeneic and the syngeneic models (Fig. 3b).

Histologically, transplanted cells displayed partial neoplastic growth on day 3, followed by substantial growth of the cells. By day 7, the typical characteristics of teratocarcinoma, including immature neural tube formation, necrosis or invasion into the surrounding tissue, were detected in the tumour, in addition to the differentiation of two or three germ-layer structures (Fig. 3a). The Ki67 index was significantly higher from day 7 onwards, compared with those on days 3 and 5 (Supplementary Fig. 1a,b, p < 0.05), and displayed a significant correlation with the SUVmax (R^2^ = 0.59, p < 0.05, Fig. 3c). The cells stained with immunohistolabeling of p53 protein, a novel oncogenic marker, presented formation of small cell clusters in the graft by day 5 and then showed rapid increase in size from day 7 onwards (Supplementary Fig. 2). As a consequence of rapid growth of tumour cells that occupyed the majority of the grafted tissue, a small number of troponin I-positive cells survived in the graft on day 7 (Supplementary Fig. 3). In contrast, the enhanced MRI and CT studies only detected the tumours on day 10, when the tumour size exceeded that of the native heart (Supplementary Fig. 4). It was, therefore, suggested that day 7 is the turning point for tumour formation in this experimental model: thus day 7 was used for the subsequent study.

### Successful manipulation of immune activity by tacrolimus injection

**Figure 4:**
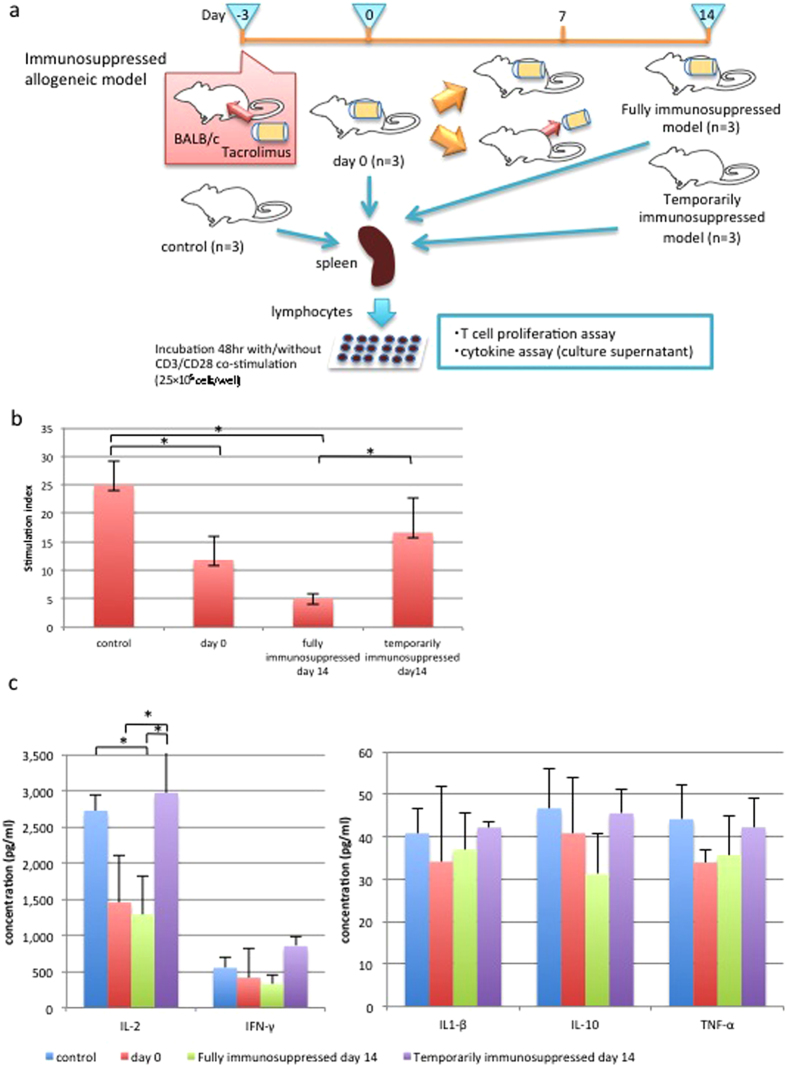
Immune competence in fully and temporarily immunosuppressed allogeneic models. (**A**) The study protocol. (**B**) Analysis of the stimulation index demonstrated that T cell proliferation was suppressed by day 14 in the fully immunosuppressed model as well as on day 0, whereas proliferation returned to normal levels by day 14 in the temporarily immunosuppressed model (n = 3; each model, *p < 0.05). (**C**) With CD3/CD28 co-stimulation, IL-2 concentrations of culture supernatant were significantly increased in control mice and in the temporarily immunosppressed mice by day 14, whereas those in the fully immunosuppressed mice displayed lower cytokine levels by day 14 (*p < 0.05). IL-1β, IL-10, TNF-α, and IFN-γ concentrations couldn’t increased with this *in vitro* assay, and there were no significant difference between these models. Abbreviations: IL; interleukin, TNF; tumour necrosis factor, IFN; interferon.

The immunological response against the teratocarcinoma was explored in the allogeneic transplantation model. BALB/c mice were divided into two experimental groups as follows: a fully immunosuppressed group in which tacrolimus infusion pumps were implanted throughout the study period and a temporarily immunosuppressed group in which the implanted tacrolimus pump was explanted on day 7 following cell-sheet transplantation (Fig. 4a).

Immunological competence in the temporarily immunosuppressed group was compared with that of the fully immunosuppressed group (n = 12). Blood tacrolimus concentrations were significantly higher in the fully immunosuppressed group on day 14, at which time tacrolimus levels were less than 1.0 ng/mL in the temporarily immunosuppressed group (p < 0.05). Additionally, splenic lymphocytes isolated from the fully immunosuppressed group displayed reduced proliferation on day 14 compared with those of control mice, and the proliferative response did not significantly differ from that on day 0. In contrast, lymphocytes isolated from the temporarily immunosuppressed group displayed a full recovery of proliferative capacity by day 14 (p < 0.05, Fig. 4b). Similarly, the interleukin (IL)-2 releasing capacity *in vitro* displayed reduced IL-2 concentration on day 0, followed by a full recovery by day 14 in the temporarily immunosuppressed group (p < 0.05), as assessed by the cytokine assay. The levels of IL-1β, IL-10, tumour necrosis factor (TNF)-α and interferon (IFN)-γ were low in the control group, and failed to reach statistically significant difference in this *in vitro* eperimental model (Fig. 4c).

**Figure 5:**
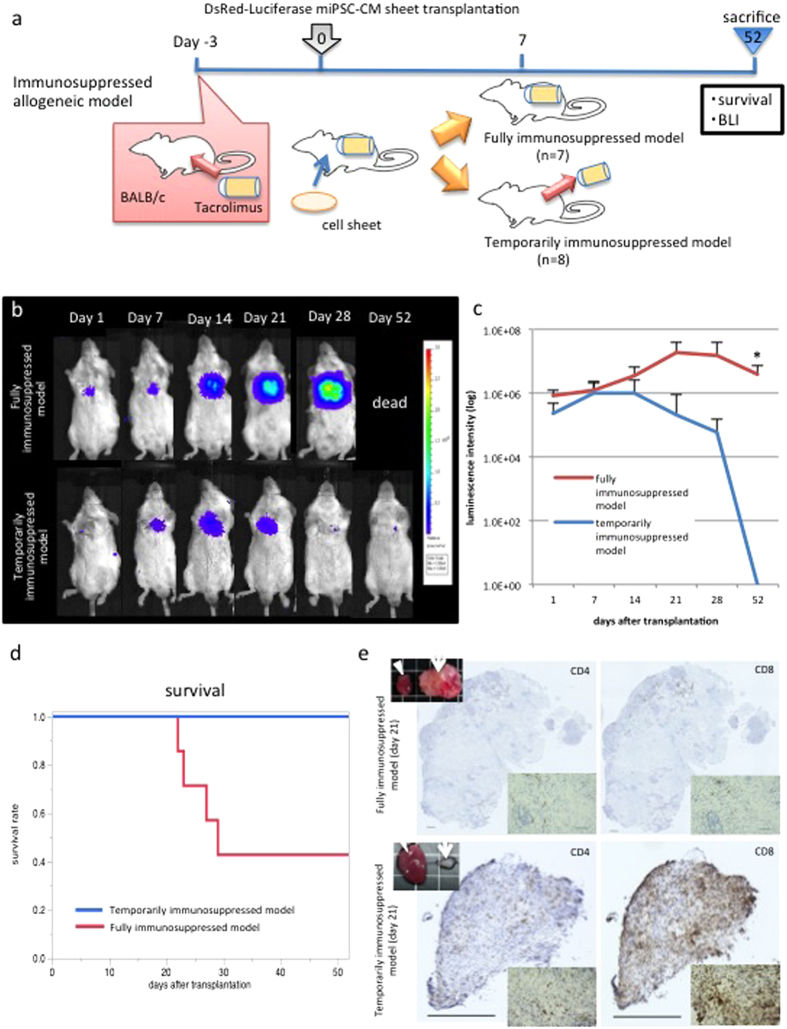
Alloimmune rejection of the teratocarcinoma after cessation of immunosuppressive therapy in temporarily immunosuppressed models. (**A**) The study protocol. (**B**) Representative serial images of BLI in one fully immunosuppressed mouse and one temporarily immunosuppressed mouse. (**C**) Quantitative analysis of BLI revealed a gradual luminescence increase in the fully immunosuppressed model (n = 7) with teratocarcinoma formation, whereas there was a gradual reduction followed by the complete elimination by day 52 in all temporarily immunosuppressed mice (n = 8) (*p < 0.05). (**D**) The temporarily immunosuppressed mice had a better survival rate than the fully immunosuppressed mice, with all temporarily immunosuppressed mice surviving the duration of the study (log rank; p < 0.05). (**E**) Immunohistolabelling of CD4/8 revealed massive T cell infiltration, particularly CD8-positive T cells into the teratocarcinoma in the temporarily immunosuppressed model (n = 6) compared with the fully immunosuppressed model (n = 3), which caused a dramatic reduction in teratocarcinoma (white arrow) compared with the host’s heart (white arrowhead) in size. Scare bars = 500 μm (left) and 100 μm (right). Abbreviation: BLI; bioluminescence imaging.

Moreover, natural killer (NK) cell activity was also examined in the control group (n = 3), fully immunosuppressed model (n = 3) and temporarily immunosuppressed model (n = 3) (Supplementary Fig. 5a). After the collection of splenic lymphocytes from each mouse, NK cells were isolated by magnetic-activated cell sorting (MACS) (Suplementary Fig. 5b), and then stimulated by plate-coated NKp46 antibody for 5 days. The rate of lysosomal-associated membrane protein-1 (LAMP-1) positive cells, which represented an activated form of NK cells, were increased in the control group and in the temporarily immunosuppressed group, however unchanged in the fully immunosuppressed group (Supplementary Fig. 5c).

### T-cell related rejection by tacrolimus cessation in allogeneic models

Teratocarcinoma formation following allogeneic transplantation of DsRed-Luciferase-miPSC-derived cardiac tissue constructs in the fully (n = 7) and temporarily immunosuppressed groups (n = 8) was serially and histologically assessed (Fig. 5a). Photons gradually increased over 28 days in six of the seven fully immunosuppressed mice (85.7%), assessed by BLI. The temporarily immunosuppressed group displayed a similar increase of photons until day 7, at which time there was a gradual reduction, followed by full elimination by day 52 (Fig. 5b,c). Although four of the seven fully immunosuppressed mice (57.1%) died by day 52 because of tumour development, all temporarily immunosuppressed mice survived until day 52 (log rank; p < 0.05, Fig. 5d). By day 52, there was no gross or histological evidence of the tumour at the cardiac surface in the temporarily immunosuppressed group.

The immunological response against the tumour was examined by immunohistolabelling of CD4 and CD8 on days 11, 14, and 21 after cell transplantation (n = 9). The accumulation of CD4 or CD8-positive cells was absent in the fully immunosuppressed group, whereas massive infiltration of CD4 and CD8-positive cells (especially CD8-positive cells) into the tumour was observed in the temporarily immunosuppressed group as early as day 11 (Fig. 5e, Supplementary Fig. 6b). Moreover, NKp46-immunohistolabeling of the tumour tissue revealed that NKp46-positive cells were minimally present in the tumour as compared to those in the spleen at any time points in the temporarily immunosuppressed model (Supplementary Fig. 6c).

**Figure 6:**
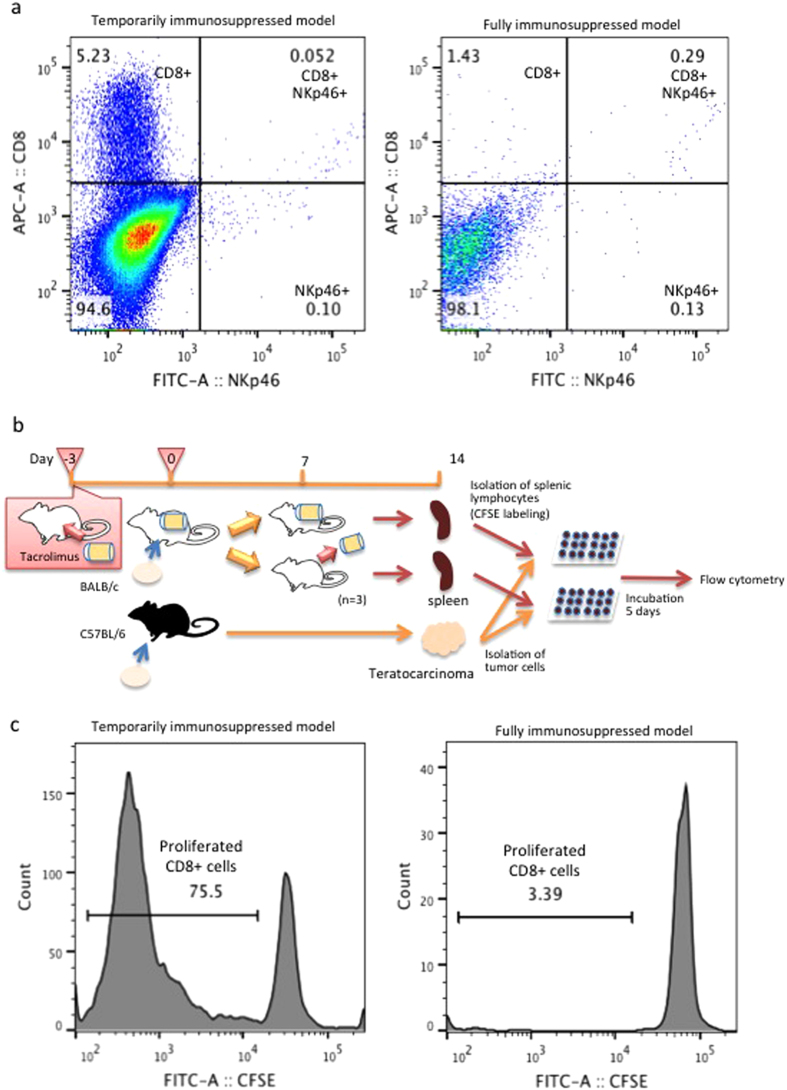
CD8-positive cells played major role of alloimmune rejection of the teratocarcinoma. (**A**) In the enzymatically dissociated cells harvested from the teratocarcinoma tissue in the temporarily immunosuppressed model on day 11 (n = 3), 5.2% of the cells were positive for CD8, whereas only 1.4% of the cells were positive for CD8 in the fully immunosuppressed models (n = 3). NKp46-positive cells were rarely detected in both of these groups. (**B**) The study protocol of MLR. (**C**) Five days after mixed incubation of splenic lymphocytes and tumour cells, cells were treated with APC-anti-mouse CD8a antibody, and CD8-positive populations were analyzed by flow cytometry. In the temporarily immunosuppressed model (n = 3), proliferated CD8-positive cells with decreased fluorescence intensity of CFSE were almost 75%, whereas those cells in the fullly immunosuppressed model were around 3%, respectively. Abbreviations: MLR; mixed lymphocytes reaction, CFSE; carboxyfluorescein succinimidyl ester.

In addition, the enzymatically dissociated cells from the tumour tissues were analyzed by flow cytometry (n = 3). In the temporarily immunosuppressed model, 5.2% of the dissociated cells were positive for CD8, whereas CD8-positive cells were only for 1.4% of the cells in the fully immunosuppressed model. There were a very small fraction of the NKp46-positive cells in both of two groups (Fig. 6a).

For the further investigation about immunological response of CD8-positive cells against the tumour cells, direct mixed lymphocytes reaction (MLR) were performed (Fig. 6b). After 5 days of mixed incubation with splenic lymphocytes and tumour cells, CD8-positive cells markedly proliferated in the temporarily immunosuppressed model, compared to in the fully immunosuppressed model (Fig. 6c). With these results considered, it was suggested that the tumour provoked T cell-related immunological response.

## Discussion

This study demonstrated that miPSC-derived cardiac tissue constructs consistently form clonal malignant teratocarcinomas within 7 days of cell-sheet transplantation in the syngeneic transplant models. The site of transplantation did not influence the teratocarcinoma formation. Continuous tacrolimus injection resulted in teratocarcinoma development in allogeneic cell-sheet transplantation model, in which the phenotype and time course was identical to those of the immunocompetent syngeneic model, as assessed by imaging tools, including ^18^F-FDG-PET. In contrast, the immunocompetent allogeneic transplantation model failed to develop teratocarcinomas. Importantly, cessation of tacrolimus injection 7 days after allogeneic cell transplantation, when malignant teratocarcinomas were present, diminished the progression of the teratocarcinoma formation in combination with accumulation of CD4 and CD8-positive T cells, resulting in the complete loss of the teratocarcinoma.

This study reported that the phenotype and time course of tumour formation from miPSC-derived cardiac tissue constructs is identical in syngeneic and immunosuppressed allogeneic cell transplantation models, suggesting that tacrolimus administration fully diminished the host-immune response against the tumour. Since tacrolimus is known to inhibit T-cell proliferation by reducing IL-2 release from the lymphocytes^11^, the tumour that formed in the presence of tacrolimus was considered to express major antigens that stimulate T cells, such as major histocompatibility complex class I. Importantly, undifferentiated iPSCs express low levels of major histocompatibility complex class I antigen, which increase following differentiation to induce a T-cell dependent immune response in allogeneic cell transplantation models^12^,^13^. In this study, a small fraction of troponin I-positive iPSC-CMs were detected in the tumour by day 7, mainly because of rapid growth of tumour cells which reduced adequate blood supply and limited the survival of iPSC-CMs, and probably because of allogeneic rejection against iPSC-CMs. Therefore, residual undifferentiated iPSCs were likely the origin of the teratocarcinomas in this study, though it was not clearly demonstrated.

There are accumulating evidence that residual undifferentiated iPSCs are the major origin of teratoma formation after transplantation therapy of derivatives of the iPSCs and should therefore be eliminated before transplantation to gurranttee the safety of the treatment^14^,^15^. In particular, genome/epigenome related mutation in the residual undifferentiated iPSCs has been considered as the major reason for teratocarcinoma formation, a malignant form of teratoma. Thus, the use of “banked” iPSCs in which genome/epigenome mutations have been fully investigated in advance would minimize the risk of teratocarcinoma formation in clinical settings, although further studies are warranted to reveal factors that exaggerate malignant tumour formation. In addition, one may propose that immunosorting-based purification of the cardiomyocytes, prior to transplantation, would minimise contamination of undifferentiated iPSCs, though this method would not fully eliminate the undifferentiated iPSCs from the graft. More importantly, purification process including enzyme based cell dissociation would impair function of the iPSC-CMs, potentially leading to inconsistent formation of the graft. Therefore, we believe that transplantation of cardiac tissue constructs derived from the banked iPSCs without cell-sorting procedure in an allogeneic manner would be the most promising approach of cardiac regenerative therapy using iPSC technology in the current clinical settings.

One may claim that the model used in this study generated excessive formation of teratocarcinoma, which would be unacceptable in the clinical settings. However, since the aim of this study was to explore immunological reaction against teratoma/teratocarcinoma of allogeneic origin, this study used the model that formed teratocarcinomas in every single animal in a consistent time-course. For this purpose, miPSC-derived cardiac tissue constucts that were comprised by a large number of iPSC-CMs contaminating with a small fraction of the undifferentiated iPSCs were transplanted over the heart surface in an allogeneic manner. Teratocarcinoma formation in this study was therefore likely to caused by intrinsic nature of the viral vector-establshed undifferentiated iPSCs of murine origin, which reportedly have abnormality or instability of the genome that would be associated with tumouringenicity.

Several imaging tools were used to explore the characteristics and the time course of tumour formation in this study. In particular, ^18^F-FDG-PET/CT scanning, which is widely used in clinical settings to visualise cells with increased glucose uptake, such as cancer cells, is reportedly useful in early diagnosis and the differential diagnosis between malignant and benign tumours^16^,^17^. In this study, glucose uptake, as assessed by ^18^F-FDG-PET scanning, was consistently upregulated in the tumour on day 7 after cell-sheet transplantation, suggesting that the tumour had acquired a malignant phenotype at least by this time-point. Histological evaluations with immunohistolabeling of Ki67 and p53 reinforced that result. In addition, the SUVmax was positively correlated with the Ki67 index, consistent with previously published studies^18^. Furthermore, the diagnosis could be made earlier by ^18^F-FDG-PET than with the other imaging tools used in this study, which essentially measure the size of the tumour. Although enhanced MRI or CT with a higher precision will allow an earlier diagnosis than those used in this study, the ^18^F-FDG-PET is indicated to be the most sensitive and reliable tool in early diagnosis of teratocarcinoma arising from iPSC-derivatives in clinical settings. A benign teratoma, which is also a critical complication in iPSC-based cell transplantation therapy, would not be diagnosed by ^18^F-FDG-PET, because glucose uptake is not substantial; thus, further studies are warranted.

In this study, cessation of immunosuppressive therapy fully diminished the teratocarcinoma in the allogeneic model. Similarly, Itakura *et al*. previously reported that cessation of immunosuppressants diminished low-grade gliomas arising from transplanted human iPSC-derived neural stem/progenitor cells in a xenotransplantation model^9^. Despite differences in the immunological responses between the allogeneic and xenogeneic transplantation models or in the characteristics of murine compared with human iPSCs, both results suggested that the immunological response could overwhelm the growth of cancer cells. Therefore, the modulation of immunosuppressants may be useful for treating teratomas/teratocarcinomas following allogeneic iPSC-CM transplantation therapy for heart disease in clinical settings.

The immunological reaction against the tumour after cessation of immunosuppressive therapy was mainly considered host T-cell related, not NK cells, in this study. As mentioned above, tacrolimus inhibits the production of cytokines, such as IL-2, to prevent clonal expansion of helper and cytotoxic T cells^11^, in concurrence with the data in this study. Moreover, Adams DH *et al*. reported that T cell migration capacity was also suppressed under the presence of tacrolimus^19^. By histological examination, massive T-cell infiltration, especially CD8-positive cytotoxic T cells, was apparent in the temporarily immunosuppressed model. Flow cytometry analysis of the enzymatically dissociated cells from the tumour tissues also revealed the presence of CD8-positive T cells in the tumour in the temporarily immunosuppressed model. More importantly, direct MLR study displayed that proliferation of splenic CD8-positive cells were markedly more substantial in the temporarily immunosuppressed group than that in the fully immunosuppressed group, suggesting that teratocarcinomas were provoled T-cell mediated host immune reaction.

Influence of NK cells on the tumour formation was also explored in this study. Firstly, effect of tacrolimus on NK cell activity, which was reportedly inconsistent^20^,^21^,^22^, was investigated *in vitro* and *in vivo* in this study. As a result, the number of NKp46-stimulated LAMP-1 positive active form of the NK cells was increased in the control and the temporarily immunosuppressed group, but not in the fully immunosuppressed group. In addition, the level of IL-2, which plays a major role for NK cell activation, was significantly smaller in the CD3/CD28 co-stimulated cells of the fully immunosuppressed group, as compared to those of the control and the temporarily immunosuppressed groups. Considering these *in vitro* study results, NK cell activity in this study might have been suppressed by the tacrolimus treatment *in vivo*. However, NKp46-positive active form of the NK cells were minimally present in the teratocarcinoma tissue in the temporarily immunosuppressed model at any time points, as compared to those in the spleen, *in vivo*. In addition, a few NK cells were detected in the enzymatically dissociated cells from the tumour tissues by flow cytometry, both in the fully and the temporarily immunosuppressed models. Although it has been reported that NK cells have a very important function in eliminating malignant tumour cells^23^, this study failed to detect the involvement of NK cell in the formation or the elimination of the teratocarcinoma *in vivo*. T cell-related response might have dominated immunological reaction against the teratocarcinoma to overwhelm the effects of the NK cells in this study using allogeneic tissue transplantation model.

This study is limited by the nature of the experimental animal model. Additionally, this study investigated only one cell line. Moreover, continuous subcutaneous tacrolimus administration using osmotic pumps displayed an inhomogeneous immunosuppressive effect in the fully immunosuppressed allogeneic model, such that one of the seven mice failed to develop a teratocarcinoma. However, the increase in the luminescence intensity of the tumours was comparable between the fully immunosuppressed and the temporarily immunosuppressed models on day 7, indicating that the main results of this study may not be affected by the consistency in the tacrolimus administration.

In conclusion, malignant teratocarcinomas arising from miPSC-derived cardiac tissue constructs provoked T cell-related host immune rejection to arrest tumour growth in a murine allogeneic transplantation model. Modulation of immunosuppressive therapy based on the time course of tumour diagnosis by ^18^F-FDG-PET successfully regulated the formation of the teratocarcinoma. Further studies are warranted to improve the diagnostic utility of ^18^F-FDG-PET and the treatment strategy against tumours for the clinical application of cell transplantation therapy using human iPSC-derived products.

## Materials and Methods

All animal experiments were performed according to the “Guide for the Care and Use of Laboratory Animals” published by the National Institutes of Health. The institutional Ethics Review Committee for Animal Experimentation approved all experimental protocols.

### Cell culture, cardiomyogenic differentiation and cell-sheet generation

DsRed-Luciferase-miPSCs were cultured in ESGRO complete PLUS Clonal Grade Medium (Merck Millipore). Cardiomyogenic differentiation was induced as previously reported without the purification process^24^,^25^,^26^. Briefly, to generate embryoid bodies (EBs), 3000 cells were dissociated in each well of a round bottom plate in the presence of 0.2 *μ*mol/L 6-bromoindirubin-3′-oxime (BIO; Merck Millipore) to activate the Wnt-signaling pathway. The effect of BIO was neutralized on day 3, and each EB was transferred to a flat bottom plate for adhesion culture on day 5. On day 14, the contracting cell clusters were dissociated, seeded on thermoresponsive dishes (5 × 10^6^ CMs/well; Upcell; CellSeed), and incubated at 37 °C for 2 days. At this time, they were then transferred to 20 °C until the cells detached spontaneously to form scaffold-free cell-sheets (Fig. 1b).

### Immunohistolabelling

After fixation with 4% paraformaldehyde phosphate buffer, the cells were treated with mouse anti-α-actinin antibody (Sigma-Aldrich), rabbit anti-troponin I antibody (Abcam), rabbit anti-firefly luciferase antibody (Abcam), and goat anti-DsRed antibody (Santa Cruz Biotechnology), subsequently treated with AlexaFluor488 goat anti-mouse IgG, AlexaFluor488 donkey anti-goat IgG, and AlexaFluor546 goat anti-rabbit IgG (Life Technologies) antibodies. The cell nuclei were counterstained with 4′, 6-diamidino-2-phenylindole dihydrochloride (DAPI). The cells were assessed using confocal laser scanning microscopy (FV 1200; Olympus).

### Semi-quantitative PCR

Total RNA was extracted and reverse-transcribed into cDNA. Semi-quantitative PCR was performed using SYBR Green and the following gene primers: glyceraldehyde-3-phosphate dehydrogenase (GAPDH), Lin28, Nanog, Oct4, ANP-1, Nkx2.5, Isl-1, and α-MHC. The sequences are summarised in Supplementary Table. All assays were performed by the Viia^TM^7 Real-Time PCR System (Life Technologies) and analysed by the relative standard curve method using the expression of GAPDH as an endogenous control.

### Flow cytometry

Cells were dissociated and treated with an anti-mouse SSEA-1 antibody (Merck Millipore) for 30 minutes, subsequently treated with a FITC-anti-mouse IgM/IgG antibody (BD) for 30 minutes. Another set of cells was fixed with CitoFix fixation buffer (BD), permeabilized with Perm/Wash buffer (BD), and treated with an anti-mouse troponin T cardiac isoform antibody (Life Technologies) for 30 minutes, followed by an APC-anti-mouse IgG antibody (BioLegend) for 30 minutes. All cells were analysed by FACS Canto II (BD).

### Cell-sheet transplantation

Adult male C57BL/6 (n = 19) and BALB/c (n = 80) mice (7–8 weeks old, 17–22 g) were generally anesthetised by inhalation of isoflurane, endotracheally intubated, and transplanted with the DsRed-Luciferase-miPSC-derived cardiac tissue construct-sheets into the left ventricular surface. The cell-sheets were also transplanted under the abdominal skin in C57BL/6 mice.

### Tacrolimus administration

An osmotic pump (ALZET Micro-Osmotic Pump model 1002; DURECT) filled with 1.5 mg/kg body weight of tacrolimus (Prograf; Astellas Pharma) was subcutaneously implanted under the back skin of the BALB/c mice (n = 71) to create an immunosuppressed allogeneic model. As one pump can continuously deliver the solution for 14 days, another new pump was added every 13 days to ensure constant release of tacrolimus. Plasma concentrations of tacrolimus were assessed by electrochemiluminescence immunoassay.

### T cell proliferation assay and cytokine assays

Splenic lymphocytes were incubated on plates coated with an anti-mouse CD3 antibody (BIOCOAT 96-well anti-mouse CD3 T cell activation plate; BD) or uncoated control plates (2.5 × 10^5^ cells/well) with 200 μl of RPMI 1640 containing 10% FBS. An anti-mouse CD28 antibody (100 ng/mL; Affymetrix) was added to each well of the CD3-coated plate. After incubation for 48 hours, 100 *μ*l of culture supernatant containing CD3/CD28 co-stimulated lymphocytes was removed and analysed by cytokine assay (Bio-Plex Pro^TM^ Mouse Cytokine 8-plex system; BIO-RAD). Ten microliters of Cell Count Regent (Nacalai Tesque) was added to each remaining 100 *μ*l of culture supernatant and the absorbances at 450 nm were measured after 2 hours. The stimulation index was calculated by dividing the absorbance of CD3/CD28 co-stimulated cells (with background adjustment) by the absorbance of unstimulated cells.

### NK cell stimulation assay

NK cells were isolated from dissociated splenic lymphocytes using mouse NK cell isolation kit (Miltenyi). Cells before and after the isolation were treated with rat anti-mouse CD49b (DX5) antibody (BioLegend), subsequently treated with FITC-anti-rat IgM antibody, and APC-anti-mouse CD3e antibody (BD), then assessed by flow cytometry. Isolated NK cells (1 × 10^5^ cells/well) were incubated on plates coated with anti-mouse CD335 (NKp46) antibody (LEAF purified anti-mouse CD335; BioLegend) for 5 days. After the incubation, cells were treated with Alexa Fluor 488 anti-mouse CD107a (LAMP-1) antibody (BioLegend) and assessed by flow cytometry.

### Tumour cell dissociation

Tumour cells were dissociated using mouse tumour dissociation kit (Miltenyi) and then treated with APC-anti-mouse CD8a antibody (BD) and FITC-anti-mouse CD335 (NKp46) antibody (BioLegend) for 30 minutes, subsequently assessed by flow cytometry.

### MLR

Splenic lymphocytes dissociated from the fully and temporarily mmunosuppressed models on day 14 were stained with carboxyfluorescein succinimidyl ester (CFSE; Cayman chemical) for 30 minutes. Teratocarcinomas were harvested from the chest cavity of C57BL/6 mice 14 days after the transplantation of cell-sheets, and tumour cells were dissociated using tumour dissociation kit. CFSE-labeled splenic lymphocytes (5 × 10^5^ cells/well) and tumour cells (5 × 10^5^ cells/well) were mixed and incubated for 5 days (Fig. 6b), then treated with APC-anti-mouse CD8a antibody (BD) and assessed by flow cytometry.

### Luciferase assay and bioluminescence imaging

The luminescence intensity of the DsRed-Luciferase-miPSC-derived cardiac tissue constructs was measured using the Luciferase Assay System (Promega) *in vitro*. BLI was performed using the IVIS Lumina II (PerkinElmer). Six minutes prior to measurement of luminescence, 150 mg/kg body weight of Rediject D-Luciferin Ultra Bioluminescence Substrate (PerkinElmer) was injected intraperitoneally. The integration time was fixed at 5 minutes for each image. All images were analyzed with Living Image Software (PerkinElmer).

### ^18^F-FDG PET, CT, and enhanced MRI studies

^18^F-FDG PET and CT scan studies were performed using the small-animal PET/CT system (Inveon; Siemens)^27^. Following an overnight fast, ^18^F-FDG (approximately 10 MBq) was injected into the tail vein. PET/CT exams were performed 60 minutes after the administration of ^18^F-FDG, and ^18^F-FDG uptake was measured for 10 minutes. The PET data were reconstructed by 256 × 256 matrix using the three-dimensional ordered subset expectation maximization method followed by maximum a posteriori (3D-OSEM/MAP; 16 subsets, 2OSEM3D, and 18 MAP iterations). Regions of interests (ROIs) were manually placed adjacent to the left ventricle with reference to the PET/CT fusion images. The SUVmax was calculated for the ROIs according to the following formula by Inveon Research Viewer software: SUV = radioactivity in ROI (Bq/cm^3^)/injected dose (Bq)/body weight (g). Enhanced MRI studies were performed by 1.5T-MRI (NEOMAX Engineering) following tail vein injection of 20 μl gadolinium (5-fold dilution) concurrently with ^18^F-FDG. Fast low angle shot sequence was used for MR acquisition (TR = 50 msec, 64 slices). All images were manipulated by OsiriX software.

### Histological analysis

Paraffin sections fixed with 4% paraformaldehyde phosphate buffer were used for hematoxylin-eosin staining and immunohistolabelling of Ki67, p53 and troponin I. Samples were microwaved in Target Retrieval Solution (pH 9.0; Dako), treated with rabbit anti-mouse Ki67 antibody (Abcam), rabbit anti-mouse p53 antibody (Leica), and rabbit anti-troponin I antibody (Abcam), followed by a horseradish peroxidase-labelled goat anti-rabbit immunoglobulin, and finally, treated with 3,3′-diaminobenzidine (DAB). The Ki67 index was calculated by dividing the number of Ki67-positive cells by the total number of tumour cells, which were observed by microscopy BZ 9000 (Keyence) and analysed by the Dynamic Cell Count function of the BZ-II Analyzer Software (Keyence) in at least 5 hot spots. Fresh frozen sections were used for immunohistolabelling of CD4, CD8 and CD335 (NKp46). Following acetone fixation, each section was treated with rat anti-mouse CD4 and CD8a antibodies (BD), followed by a horseradish peroxidase-labelled polymer, and then exposed to the DAB color development solution. For the immunohistolabeling of CD335, each section was acetone fixated, treated with rat anti-mouse CD335 antibody (BioLegend), and subsequently treated with FITC-anti-rat IgG antibody.

### Statistical analysis

Continuous variables were reported as mean ± standard deviation and were compared using Student’s t-test or one-way analysis of variance with Tukey’s honestly significant difference test. P-values < 0.05 were considered statistically significant. Correlations between cell count and luminescence intensity by *in vitro* luciferase assay or SUV max and Ki67 index in the ^18^F-FDG-PET studies were analysed in pairs using Pearson’s correlation analysis. Survival rates of mice were demonstrated by Kaplan-Meier curve and compared by log-rank test. All statistical analyses were performed using JMP 11.0 (SAS).

## Additional Information

**How to cite this article**: Kawamura, A. *et al*. Teratocarcinomas Arising from Allogeneic Induced Pluripotent Stem Cell-Derived Cardiac Tissue Constructs Provoked Host Immune Rejection in Mice. *Sci. Rep*. **6**, 19464; doi: 10.1038/srep19464 (2016).

## Supplementary Material

Supplementary Information
