# Supplementary material for: Teratocarcinomas Arising from Allogeneic Induced Pluripotent Stem Cell-Derived Cardiac Tissue Constructs Provoked Host Immune Rejection in Mice
Source: Sci Rep. 2016 Jan 14;6:19464. doi: 10.1038/srep19464 (PMC4725880; doi:10.1038/srep19464)
Supplement: Supplementary Information [file srep19464-s1.doc]

**Supplementary Information**

**Teratocarcinomas Arising from Allogeneic Induced Pluripotent Stem Cell-Derived Cardiac Tissue Constructs Provoked Host Immune Rejection in Mice**

Ai Kawamura, Shigeru Miyagawa, Satsuki Fukushima, Takuji Kawamura, Noriyuki Kashiyama, Emiko Ito, Tadashi Watabe, Shigeo Masuda, Koichi Toda, Jun Hatazawa, Eiichi Morii, Yoshiki Sawa

**Supplementary Table:** The sequence of primers used by semi-quantitative reverse transcriptional PCR.

| **primer** | **sequence** |
| --- | --- |
| **GAPDH (forward)** | 3'-CCAGTATGACTCCACTCACG-5' |
| **GAPDH (reverse)** | 5'-GACTCCACGACATACTCAGC-3' |
| **Lin28 (forward)** | 3'-CTGCTGTAGCGTGATGGTTGA-5' |
| **Lin28 (reverse)** | 5'-CCACCCAATGTGTTCTATTGCA-3' |
| **Nanog (forward)** | 3'-TCGCCATCACACTGACATGA-5' |
| **Nanog (reverse)** | 5'-TGTGCAGAGCATCTCAGTAGCA-3' |
| **Oct4 (forward)** | 3'-TTTAACCCCAAAGCTCCAGG-5' |
| **Oct4 (reverse)** | 5'-GGCTCTCCCATGCATTCAA-3' |
| **ANP-1 (forward)** | 3'-AAAGAAACCAGAGTGGGCAGAG-5' |
| **ANP-1 (reverse)** | 5'-CCAGGGTGATGGAGAAGGAG-3' |
| **Nkx2.5 (forward)** | 3'-CAAGTGCTCTCCTGCTTTCC-5' |
| **Nkx2.5 (reverse)** | 5'-GGCTTTGTCCAGCTCCACT-3' |
| **Isl-1 (forward)** | 3'-TTTCCCTGTGTGTTGGTTGC -5' |
| **Isl-1 (reverse)** | 5'-TGATTACACTCCGCACATTTCA-3' |
| **α-MHC (forward)** | 3'-GAGATTTCTCCAACCCAG-5' |
| **α-MHC (reverse)** | 5'-CCAGGGTGATGGAGAAGGAG-3' |

**Supplementary Figure 1: Immunohistolabeling of Ki67 in the syngeneic teratocarcinomas.**

**
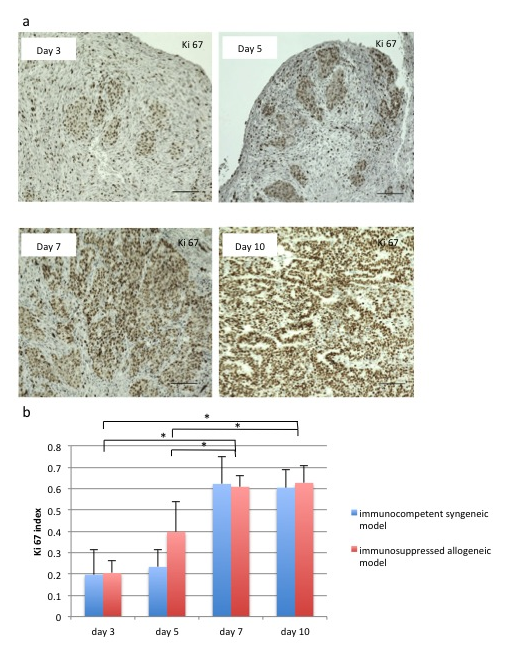
**

(A) Representative images with immunohistolabeling of Ki67 protein in the teratocarcinoma cells (immunocompetent syngeneic model). Scale bars = 100 μm.

(B) The Ki67 index demonstrated significantly higher values from day 7 onwards, compared with those on days 3 and 5 (* p < 0.05).

**Supplementary Figure 2: Immunohistolabeling of p53 in the syngeneic teratocarcinomas.**

**
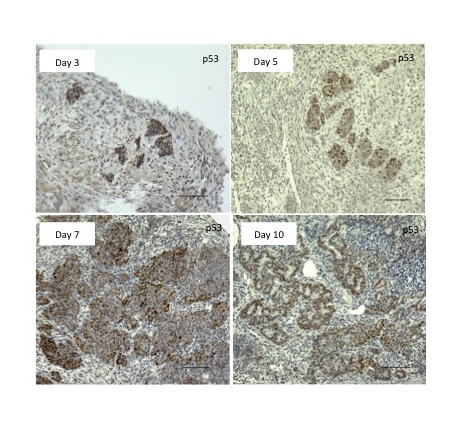
**

Immunohistolabeling of p53 protein demonstrated that cells with positive for p53 protein in nuclei increased rapidly from day 7 onwards, which accorded with the results of immunohistolabeling of Ki67, suggesting that tumours acquired malignant phenotype from day 7 onwards.

Scale bars = 100 μm.

**Supplementary Figure 3: Immunohistolabeling of troponin I in the teratocarcinoma.**


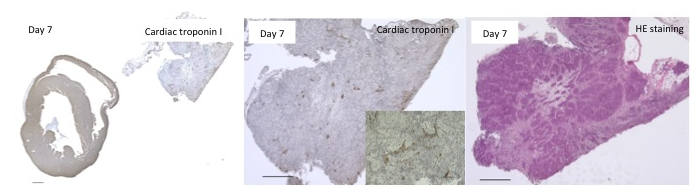


Immunohistolabeling of troponin I revealed that there remained a small number of troponin I-positive cells surrounding to the teratocarcinoma cells on day 7 after cell-sheet transplantation.

Scale bars = 500 μm (left) and 100 μm (right).

Abbreviation: HE; hematoxylin-eosin.

**Supplementary Figure 4: Enhanced MRI and plain CT images in the syngeneic model.**


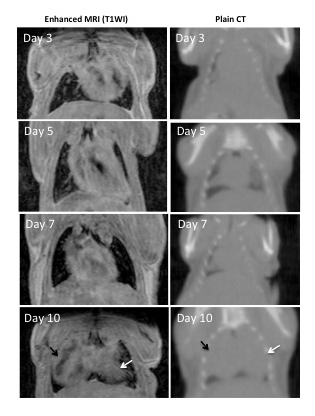


Representative serial images of enhanced MRI (T1WI) and plain CT studies are presented. The teratocarcinoma (white arrow) was detected only on day 10, when the tumour exceeded the host’s heart (black arrow) in size and displaced the heart to the right side.

Abbreviations: MRI; magnetic resonance imaging, T1WI; T1 weighted image, CT; computed tomography.

**Supplementary Figure 5: NK cell stimulation assay in the fully and temporarily immunosuppressed models.**

**
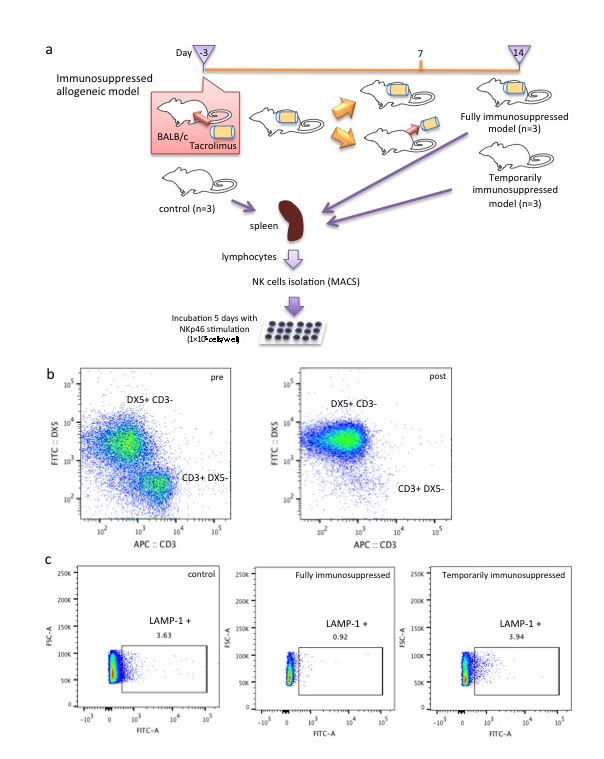
**

(A) Study protocol.

(B) After the isolation of NK cells, CD3 positive cells was depleted and almost all cells were positive for DX5, suggesting the successful isolation of NK cells.

(C) With NKp46 stimulation for 5 days, LAMP-1 positive cells, an active form of NK cells, increased in the control group (n=3) and in the temporarily immunosuppressed model (n=3), whereas those cells didn’t increased in the fully immunosuppressed model (n=3).

Abbreviations: MACS; magnetic-activated cell sorting, LAMP-1; lysoso,al-associated membrane protein-1.

**Supplementary Figure 6: The time course of T cell infiltration and NK cell presence into the teratocarcinomas in the temporarily immunosuppressed model.**


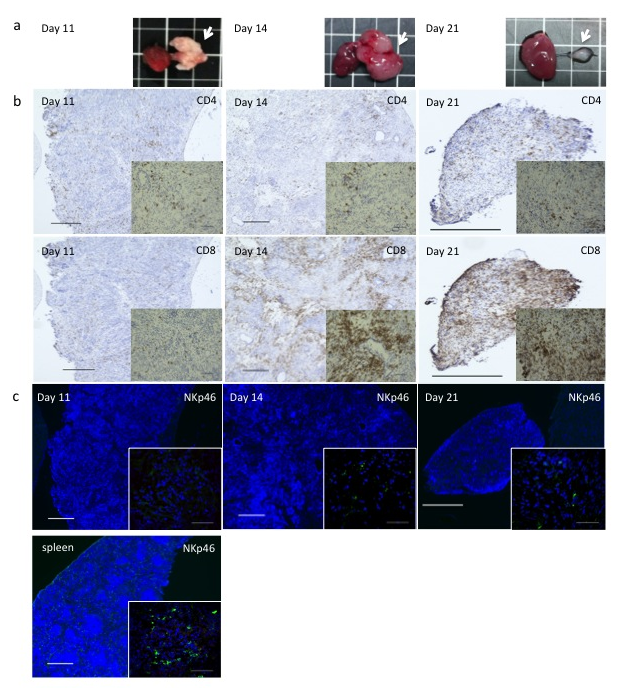


(A) Representative images of teratocaricinomas (white arrow) revealed that tumours grossly decreased in size 14 days after cessation of immunosuppressive therapy in the temporarily immunosuppressed model (day21).

(B) Immunohistolabeling of CD4/CD8 demonstrated that accumulating T cell infiltration in the teratocarcinomas, especially CD8-positive cells, were apparent with the time course.

(C) Immunohistolabeling of NKp46 demonstrated that few NK cells were detectable in the teratocarcinomas through the time course, as compared to those in the spleen tissue.

Scale bars = 500 μm (left) and 100 μm (right).
